# Assessing randomness and complexity in human motion trajectories through analysis of symbolic sequences

**DOI:** 10.3389/fnhum.2014.00168

**Published:** 2014-03-31

**Authors:** Zhen Peng, Tim Genewein, Daniel A. Braun

**Affiliations:** ^1^Max Planck Institute for Biological CyberneticsTübingen, Germany; ^2^Max Planck Institute for Intelligent SystemsTübingen, Germany; ^3^Graduate Training Centre of NeuroscienceTübingen, Germany

**Keywords:** motion randomness, motion complexity, Lempel-Ziv complexity, approximate entropy, conditional entropy, effective measure complexity, excess entropy

## Abstract

Complexity is a hallmark of intelligent behavior consisting both of regular patterns and random variation. To quantitatively assess the complexity and randomness of human motion, we designed a motor task in which we translated subjects' motion trajectories into strings of symbol sequences. In the first part of the experiment participants were asked to perform self-paced movements to create repetitive patterns, copy pre-specified letter sequences, and generate random movements. To investigate whether the degree of randomness can be manipulated, in the second part of the experiment participants were asked to perform unpredictable movements in the context of a pursuit game, where they received feedback from an online Bayesian predictor guessing their next move. We analyzed symbol sequences representing subjects' motion trajectories with five common complexity measures: predictability, compressibility, approximate entropy, Lempel-Ziv complexity, as well as effective measure complexity. We found that subjects' self-created patterns were the most complex, followed by drawing movements of letters and self-paced random motion. We also found that participants could change the randomness of their behavior depending on context and feedback. Our results suggest that humans can adjust both complexity and regularity in different movement types and contexts and that this can be assessed with information-theoretic measures of the symbolic sequences generated from movement trajectories.

## Introduction

Imagine you were abandoned on an uninhabited planet and you could move around on the surface, thereby generating motion trajectories that can be observed by some non-human intelligence. How would you move in a way to show that you are an intelligent being? Or as an observer, what trajectories of moving bacteria would convince you that you are observing an intelligent organism? Similar questions that are not necessarily confined to motion trajectories are considered by space programs such as SETI (search for extraterrestrial intelligence), with the idea that intelligence should be related to behavioral complexity.

Previous studies have used the concept of Kolmogorov complexity, for example, to evaluate the complexity of animal behavioral patterns, such as ants' hunting behavior (Panteleeva et al., 2010; Reznikova et al., 2012). In these studies the authors assessed the regularity of behavioral sequences and found that successful hunting behavior was associated with higher stereotypy. The regularity of single joint movements has also been studied in humans (Newell et al., 2000). In this study the authors found that humans can generate only very limited randomness and that they cannot substantially increase the degree of motion randomness through training. In contrast, behavioral studies in psychology have indicated that the randomness of human-generated random number sequences might be dependent on the feedback provided to human subjects (Neuringer, 1986; Persaud, 2005; Figurska et al., 2008).

Measures, such as Kolmogorov complexity, might seem to suggest that complexity can be measured by the degree of irregularity or randomness. Kolmogorov complexity is the length of the shortest program that can generate a certain symbolic sequence (Kolmogorov, 1963). Therefore, sequences that can be described by a short program have low complexity, because their information can be compressed into a shorter description. In contrast, complex sequences are incompressible. For example, a binary sequence generated by a fair coin would be the most complex sequence, as its shortest description is simply a copy of the random sequence itself. Yet, intuitively, we feel that such a sequence is not very complex and in fact rather simple to generate. An intuitive example with high complexity is human language, where one typically finds that sequences of letters or words are neither completely random nor totally determinate. This is often assessed quantitatively by studying the conditional entropy of sequences (Rao et al., 2009). The conditional entropy quantifies the degree of uncertainty about the next word or symbol conditioned on a history of symbols. If this uncertainty shrinks over long histories, this implies that there are long range correlations that reflect an underlying complex structure.

In our study we address two questions. First: How can we measure human motion complexity? To this end, we quantify both regularity and organizational complexity by determining predictability, compressibility, approximate entropy, Lempel-Ziv complexity and effective measure complexity of different movement types. Second: Can humans change the degree of motion randomness depending on feedback and context? To answer this question, we designed a pursuit game in which we could compare subjects evasive random motion under feedback to their previous self-paced random motion in the absence of feedback.

## Results

**Figure 1:**
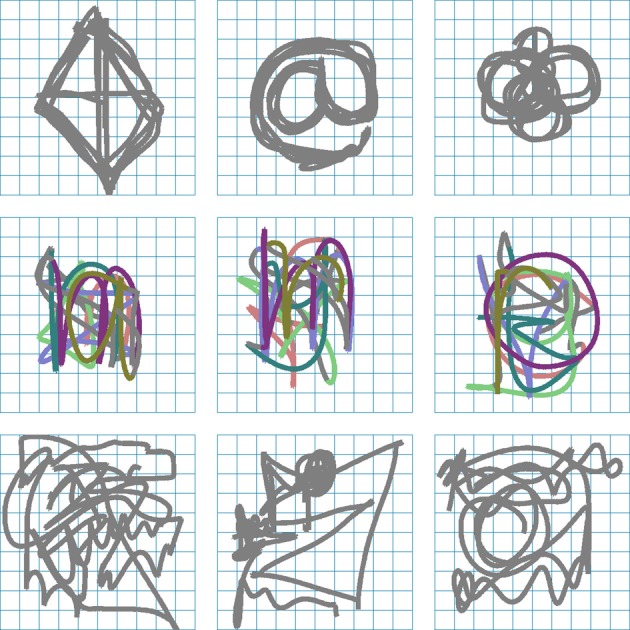
**Example trajectories**. The top row shows three examples of patterns freely generated by subjects **(P)**, the middle row shows three examples of letter sequences **(L)**, and the bottom row shows three self-paced random trajectories **(R1)**. The different colors in the middle row segment the individual letters superimposed on each other and are for illustrative purposes only.

Participants controlled a cursor in a three-dimensional virtual environment consisting of 10 × 10 grid cells. In the first part of the experiment participants were asked to generate repetitive *patterns* (**P**) freely, write pre-specified letters (**L**), and perform self-paced random movements (**R1**). Figure 1 shows recorded example trajectories drawn by subjects in the experiment. The first row of panels shows subjects' self-created patterns that could contain both relatively complicated repetitive structures—for example, a repetitive drawing of the “@”-symbol—and simpler structures like circles or squares. The second row shows examples of drawn letter sequences, where different letters are drawn on top of each other and the different colors illustrate individual letter segments. The bottom row of panels shows examples of self-paced random movements. Upon inspection these trajectories contained no obvious global structure.

All movements were converted into symbol sequences of up, down, left, and right cell transitions. This discretization is a standard procedure in the mathematical field of symbolic dynamics to model a smooth dynamical system through a finite cover, thus allowing to represent the history and future of the system by strings of symbols. Estimating the entropy of such symbol strings with arbitrarily chosen discretization always provides a lower bound on the entropy of the underlying smooth system (Badii and Politi, 1999). In order to have a baseline comparison, we also generated artificial data from three synthetic processes. One process generated simple artificial rhythmic movements (**AS**) consisting of a completely regular repetition of up-down transitions, another process generated artificial random movements (**AR**) following a random walk in a 10 × 10 grid, and the last process generated artificial random movements having the same first order frequencies as the subjects' pattern generation sequences (**AF**).

### Measures of motion regularity

To quantify the degree of regularity in symbol sequences generated by different movement types, we used four different measures. First, we determined the *predictability* of movements with the idea that the more regular a movement is, the easier it is to predict. We used a Bayesian predictor that could track histories of up to 8 cell transitions to make predictions about the next move—see *Context-tree weighting* algorithm in the Methods. Second, we determined the *compressibility* of movements, again with the idea that the more regular a movement is, the easier it is to exploit patterns for compression. Since any probabilistic predictor can always be used for compression, we used our Bayesian predictor also as a compressor. Additionally, we also used standard Lempel-Ziv compression to assess regularity in the movement data. As a third measure of regularity, we determined the *approximate entropy* (ApEn) of movements. The greater the value of ApEn, the higher the irregularity of the time series, thus the more complex the system under study (Pincus, 1991, 1995). Fourth, we computed the *Lempel-Ziv complexity*, that is the number of words in a dictionary required to express a symbol string without losing information (Doğanaksoy and Göloğlu, 2006). The size of the dictionary depends of course on the regularity in the string, such that the more regular the sequence, the smaller the dictionary.

**Figure 2:**
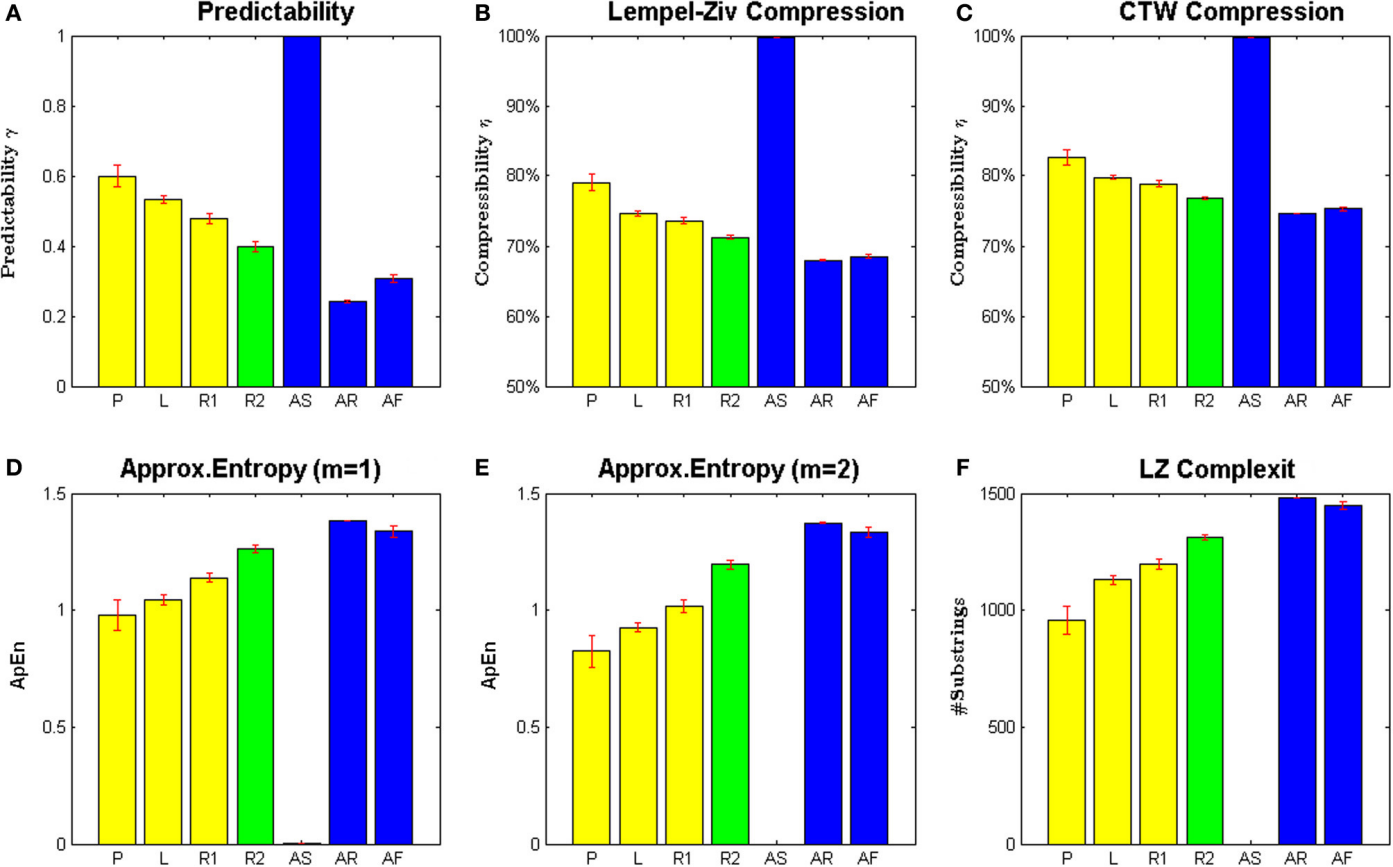
**Measures of regularity of different motion types. (A)** Shows predictability of the next cell transition given a Bayesian context-tree weighting predictor. **(B,C)** Show compressibility of cell transition sequences based on Lempel-Ziv compression and arithmetic coding of context-tree weighting probabilities. **(D,E)** Show approximate entropies for two different parameter settings. **(F)** Shows Lempel Ziv complexity of the different motion types. All bars show means and standard error over subjects. The yellow bars show values of self-paced movements from the *drawing session*, including self-generated patterns **(P)**, letter drawings **(L)**, and random motion **(R1)**. The green bars show values of random motion during the *pursuit game* **(R2)**. The blue bars show artificially generated data (**AS, AR**, and **AF**).

The results of these analyses can be seen in Figure 2 and Table 1. All panels show the average value over 10 subjects, and error bars indicate standard errors. Figure 2A shows the predictability of each movement type given by the proportion of moves that could be correctly predicted by our Bayesian model. It can be seen that the extremes are spanned by the three synthetic processes. The artificial random movements (**AR** and **AF**) are most difficult to predict, whereas the artificial rhythmic movement (**AS**) is completely predictable. Subjects' movements are in between these extremes, where the self-generated patterns (**P**) were the most predictable, followed by drawn letters (**L**). Subjects' random movements (**R1**) were most difficult to predict within the set of self-paced motions, but significantly easier to predict than artificial random motions (**AR** and **AF**).

**Table 1:** **Effect of the different movement types on the proposed measures of motion regularity and complexity**.

**Measures**	**Effect across conditions**	**P–L**	**P–R1**	**P–R2**	**L–R1**	**L–R2**	**R1–R2**
Predictability	^***^	^*^	^**^	^**^	^**^	^**^	^**^
LZ compression	^***^	^**^	^**^	^**^	−	^**^	^**^
CTW compression	^***^	^*^	^**^	^**^	−	^**^	^**^
ApEn (*m* = 1)	^***^	−	^*^	^**^	^*^	^**^	^**^
ApEn (*m* = 2)	^***^	−	^*^	^**^	^*^	^**^	^**^
LZ complexity	^***^	^*^	^**^	^**^	^*^	^**^	^**^
EMC	^***^	^**^	^**^	^**^	^*^	^**^	−

*We ran a permutation ANOVA with 2000 simulations (Anderson and Ter Braak, 2003). We found the main effect that all measures differed significantly for the different movement types. We then applied Bonferroni-Holm correction methods (Holm, 1979) for multiple post-hoc pairwise comparisons. Three asterisks indicate a significance level of p < 0.001, two asterisks indicate p < 0.01, and one asterisk indicates p < 0.05*.

Figures 2B,C show the results of the compression analysis. It can be seen that both compression methods reveal the same ranking with respect to regularity of the various movement types. The fact that the Bayesian compressor achieves generally better compression is not important, since only the relative differences between different movement types matter. Again the extremes are spanned by the three synthetic processes. The artificial random processes (**AR** and **AF**) are most difficult to compress, while the artificial rhythmic process (**AS**) is easiest to compress. In the set of subjects' movements, self-generated patterns (**P**) have the highest compressibility, suggesting the presence of structure and regularity. Subjects' random movements (**R1**) have the lowest compressibility, but are clearly more compressible than artificial random motions (**AR** and **AF**). The compressibility of letter drawings (**L**) is very similar to the compressibility of subject's self-paced random motion (**R1**).

Figures 2D,E show the approximate entropy (ApEn) for the different movement types, again with the highest irregularity in artificial random data (**AR** and **AF**), followed by subjects' random movements (**R1**), the letter drawings (**L**) and subjects' self-generated patterns (**P**). The artificial rhythmic movement (**AS**) had an approximate entropy of zero. We found the value of the approximate entropies to be robust with respect to the parameter range used by previous studies—see Methods for details.

Figure 2F shows the Lempel-Ziv complexity for the different movement types, again with the highest irregularity in artificial random data (**AR** and **AF**), followed by subjects' random movements (**R1**), the letter drawings (**L**), and subjects' self-generated patterns (**P**). Importantly, all four measures indicate the same order of regularity in our movement data, which suggests that they provide a robust measure to assess regularity and randomness in motion trajectories.

### Adaptation of motion regularity during pursuit

To investigate whether the degree of irregularity that subjects can generate during self-paced random motion can be modified, we designed a second part of the experiment where participants were asked to perform unpredictable movements in the context of a pursuit game. The aim of the game was to avoid being caught by a pursuer that predicted subjects' next move.

**Figure 3:**
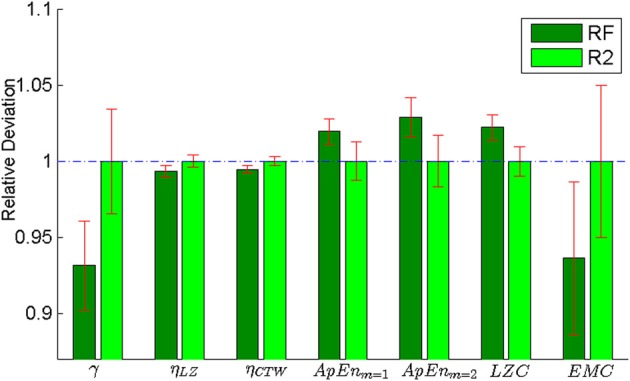
**Comparison between online (RF) and offline feedback (R2) in the pursuit game**. The plot shows the results of all the measures considered in this paper: predictability, compressibility using a Lempel-Ziv compressor, compressibility using a Context-Tree Weighting compressor, Approximate Entropy with two different parameter settings (*m* = 1 and *m* = 2), Lempel-Ziv Complexity and Effective Measure Complexity. All values are normalized with respect to the offline condition **(R2)**. We found no significant difference in irregularity or structural complexity between online and offline feedback trials (see Table 2 for detailed results).

The pursuer was simulated by an adaptive Bayesian model that predicted subjects' next cell transition and that could learn subjects' idiosyncrasies over trials. Subjects received feedback about the pursuer's success either in an online fashion during the trial or in an offline fashion after each trial. In online feedback trials, cells were colored red and a beep sound occurred whenever subjects' cell transition was predicted by the Bayesian model. In offline feedback trials, the proportion of correctly predicted moves was displayed to subjects after trial completion. Both trial types were intermixed randomly. We found no significant difference in irregularity or structural complexity between online and offline feedback trials—see Figure 3 and Table 2 for detailed results. The rationale for the two trial types was that the online feedback condition (**RF**) served mainly as a learning condition, whereas the task setup for the offline feedback condition (**R2**) was comparable to the self-paced random motion condition (**R1**), since in either case there was no performance feedback during the trial. Therefore, we only show results for the offline feedback condition in Figure 2.

**Table 2:** **Comparision between online (RF) and offline feedback (R2) in the pursuit game**.

**Measures**	***p*-value**	**Sign. level**
Predictability	0.146	−
LZ compression	0.238	−
CTW compression	0.163	−
ApEn (*m* = 1)	0.218	−
ApEn (*m* = 2)	0.174	−
LZ complexity	0.101	−
EMC	0.377	−

*We ran a permutation ANOVA with 2000 simulations on the proposed measures of motion regularity and complexity. We found no signifficant difference in irregularity or structural complexity between online and offline feedback trials*.

As can be seen in Figure 2, the irregularity of random movements in the pursuit game was increased compared to self-paced random motion. This increase was statistically significant for all considered randomness measures—compare Table 1. However, the generated random trajectories even after training in a pursuit game were still not as random as Brownian motion (*p* < 0.001, Mann–Whitney–Wilcoxon test with Bonferroni-Holm correction) or a first-order Markov process based on subjects' empirical frequencies (*p* < 0.05).

### Complexity vs. randomness

When applying the previous measures of predictability, compressibility, approximate entropy, and Lempel-Ziv complexity as shown in Figure 2, we see that the highest degree of irregularity is always achieved by artificial random trajectories. However, irregularity itself is not necessarily a measure of complexity, but rather of randomness, and randomness in turn might be generated by quite simple processes—e.g., by flipping a fair coin. In contrast, one would feel intuitively that a complex motion should lie somewhere in between the two extremes of completely predictable regularity and structureless randomness.

**Figure 4:**
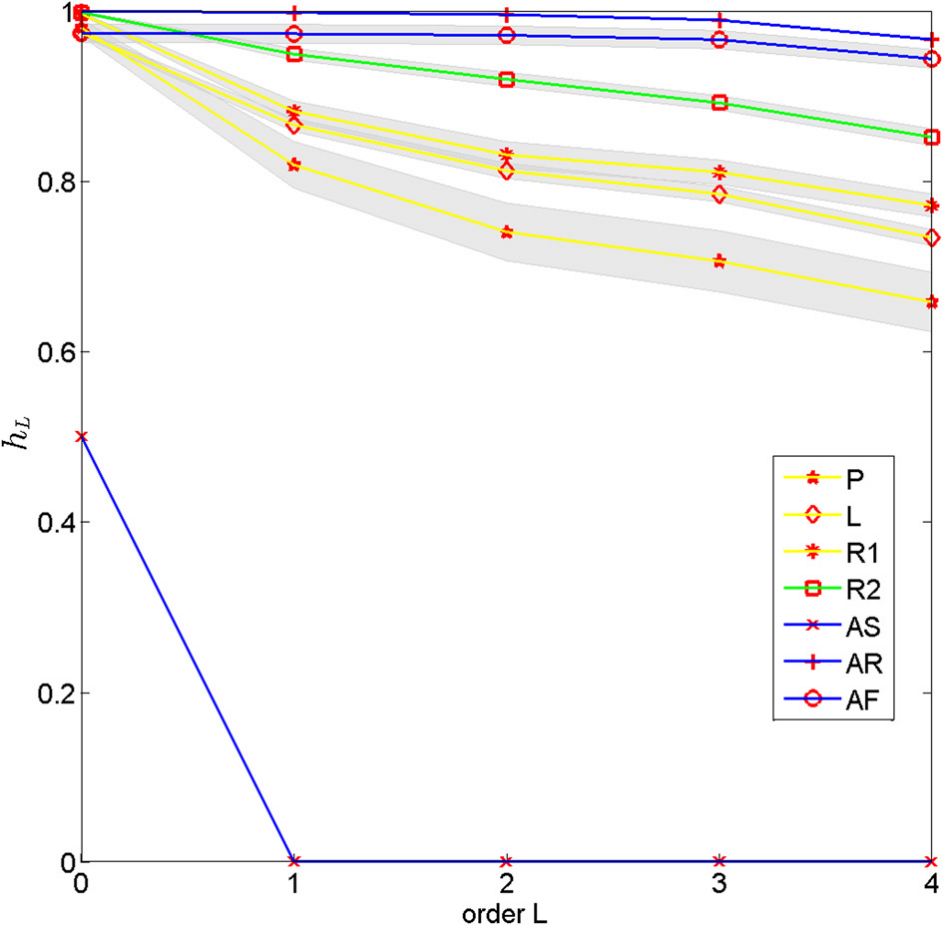
**Conditional entropy for different movement types estimated from empirical frequencies depending on the length of the history**. The different movement types included self-generated patterns **(P)**, letter drawings **(L)**, self-paced random motion **(R1)**, random motion during pursuit **(R2)** and artificial rhythmic **(AS)** and random motions (**AR** with uniform distribution and **AF** with empirical first order frequency). The lines show means over subjects and the gray bands show standard errors.

A good starting point to assess the degree of organizational complexity is to analyze conditional entropies of a process. The conditional entropy quantifies the degree of uncertainty about the next state of the process conditioned on a history of states. In particular, if conditional entropies are sensitive to long histories, this suggests that there are long-range correlations and structure typical for complex processes. To assess the complexity of subjects' motion trajectories quantitatively we therefore investigated the dependence of conditional entropy on history length. Figure 4 compares the conditional entropy estimated from empirical frequencies up to length 4 for the different movements types.

The conditional entropy for the completely predictable artificial rhythmic movement (**AS**) immediately decays to zero once the history is long enough to include the repetitive pattern. In contrast, the conditional entropy of the artificial random movements (**AR** and **AF**) shows almost no decay—there is only a tiny decay due to boundary effects of the 10 × 10 grid—, because the entropy in each time step conditioned on any history remains (almost) the same. When comparing the different human movements to each other, one can see that the conditional entropy of self-generated motion patterns (**P**) decays the fastest, followed by the conditional entropy of the letter drawings (**L**), indicating the fast increase in predictability when including longer motion histories. When comparing the two random motions, one can see that the conditional entropy in the pursuit game (**R2**) decays slower than the self-paced random motion (**R1**), suggesting that subjects were able to reduce temporal correlations in their movements.

**Figure 5:**
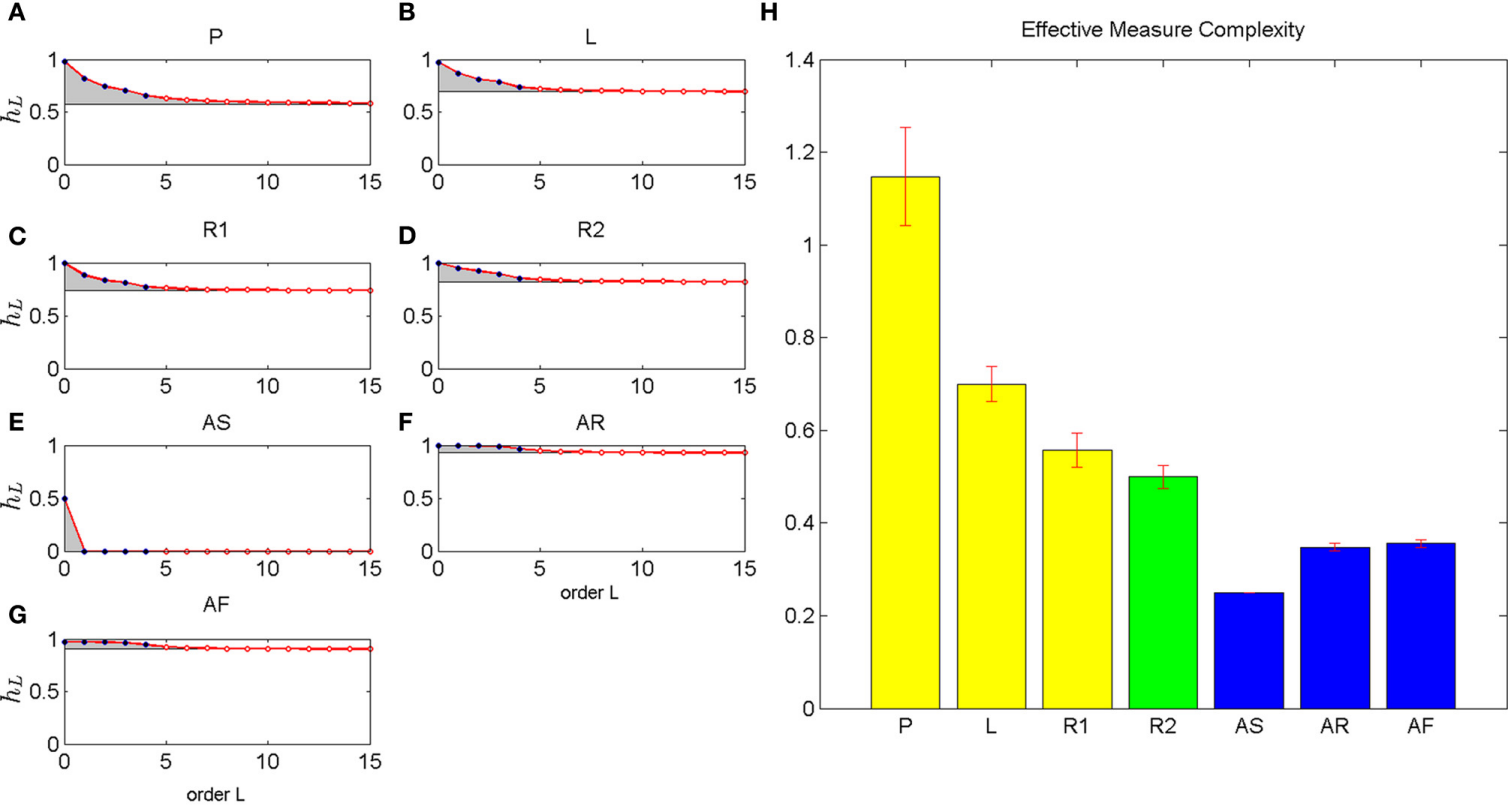
**Estimated conditional entropy and effective measure complexity**. The left six panels **(A–G)** shows the estimated conditional entropy for different movement types. Up to order 4 the conditional entropy is estimated from empirical frequencies (blue dots). The asymptote (black line) is estimated from LZ complexity. The red dots show a parametric interpolation of conditional entropies according to Equation 9. The gray areas indicate the integrated excess entropies that yield the effective measure complexity shown in the right panel **(H)**. The different movement types included self-generated patterns **(P)**, letter drawings **(L)**, self-paced random motion **(R1)**, random motion during pursuit **(R2)**, and artificial rhythmic **(AS)** and random motions (**AR** with uniform distribution and **AF** with empirical first order frequency).

It is notoriously difficult to obtain reliable estimates of higher order conditional entropies from finite data, since the number of potential histories grows exponentially. However, it is possible to estimate the asymptotic value of the conditional entropy, which is called the *entropy rate*. The entropy rate quantifies the irreducible part of the uncertainty of a stochastic process that cannot be further reduced by taking into account longer histories (Prokopenko et al., 2009). We can estimate this entropy rate from finite data by computing the normalized Lempel-Ziv complexity. The resulting estimates of the entropy rates for the different movement conditions are drawn as asymptotes in Figure 5.

Importantly, we can think about the conditional entropies as approximations to the entropy rate when we condition on finite histories rather than infinitely long histories. These finite history approximations systematically overestimate the entropy rate, because taking into account more information in the history can only improve prediction. Therefore, the systematic overestimation quantifies the part of the randomness that vanishes when considering longer histories for prediction, and is therefore not really randomness at all, but an indication of structure. The total complexity of the structure can then be obtained by the *effective measure complexity* that integrates the differences between finite history conditional entropies and entropy rate for all possible history lengths—see Methods for details.

To obtain an estimate of the effective measure complexity, we assumed a parametric form (Ebeling and Nicolis, 1991, 1992) for the decay of conditional entropies for histories longer than order 4 that interpolated between the empirical conditional entropies up to order 4 and the asymptotic estimates given by the normalized Lempel-Ziv complexity (Lempel and Ziv, 1976; Cover and Thomas, 1991; Badii and Politi, 1999). The gray areas in Figure 5 show the integral of the differences between the finite history conditional entropies and entropy rates. This integral defines our estimate of the effective measure complexity.

In contrast with the previous measures of regularity, the artificial rhythmic (**AS**) and both artificial random movements (**AR** and **AF**) have lower complexity than any movements generated by humans. The highest complexity is obtained by subjects' self-generated patterns (**P**), followed by subjects' drawing of letter sequences (**L**). The lowest complexity among subjects' movements is seen for subjects' random movements (**R1** and **R2**).

### Influence of showing trajectories

In the previous experiment, we showed subjects the entire history of their movement trajectories during each trial. However, this may add some external memory and auto-information structure in the joint system of human and virtual environment. In order to study the impact of displaying trajectories, we conducted a control experiment with another 10 subjects. The control experiment followed exactly the same procedure as the previous experiment, with the only difference that the history of movement trajectories were not shown to subjects, but only the current hand position.

**Figure 6:**
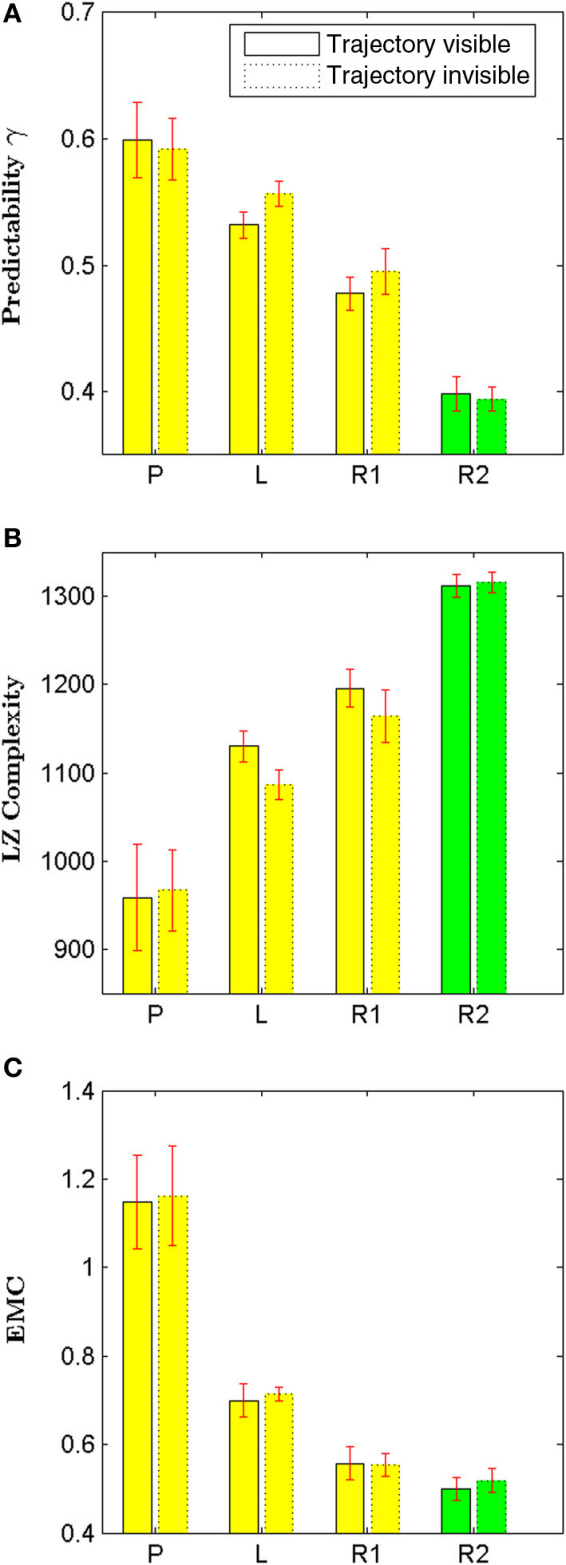
**Effect of showing participants the entire history of their movement trajectories**. We compare four different types of movement: self-generated patterns **(P)**, letter drawings **(L)**, self-paced random motion **(R1)**, and random motion during pursuit **(R2)** using three different measures: predictability in **(A)**, Lempel Ziv complexity in **(B)**, and Effective Measure Complexity in **(C)**. We found no significant difference for any of these measures when comparing both conditions (see Table 3 for detailed results).

Figure 6 compares the results obtained from the control experiment with the results obtained from the original experiment. We found that neither irregularity nor structural complexity measurements changed significantly for all movement types—see Table 3 for detailed results. This suggests that our results are not an artifact of displaying the trajectory history, but hold more generally. In particular, we found that the relative order of regularity and complexity of different movement types remained the same as in the original experiment.

**Table 3:** **Effect of displaying trajectory trace**.

**Measures**	**P–P**		**L–L**		**R1–R1**		**R2–R2**	
	***p*-value**	**Sign. level**	***p*-value**	**Sign. level**	***p*-value**	**Sign. level**	***p*-value**	**Sign. level**
Predictability	0.396	−	0.121	−	0.312	−	0.367	−
LZ compression	0.425	−	0.106	−	0.312	−	0.425	−
CTW compression	0.367	−	0.061	−	0.339	−	0.192	−
ApEn (*m* = 1)	0.425	−	0.093	−	0.396	−	0.367	−
ApEn (*m* = 2)	0.312	−	0.093	−	0.367	−	0.214	−
LZ complexity	0.396	−	0.070	−	0.339	−	0.214	−
EMC	0.440	−	0.325	−	0.396	−	0.339	−

*We ran Mann–Whitney–Wilcoxon text to study the effect of showing or not showing the entire history of their movement trajectories. We found no significant difference for any of the proposed measures of motion regularity and complexity when comparing both conditions*.

### Effect of grid size

**Figure 7:**
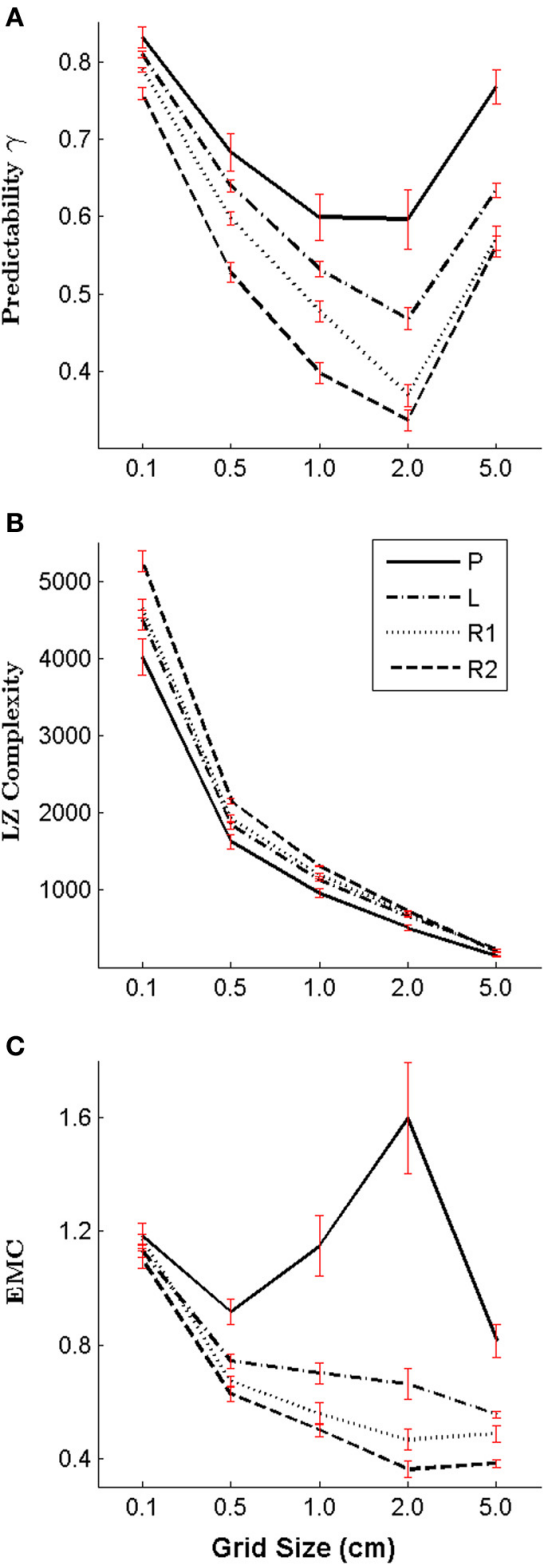
**Impact of the grid size on the regularity and complexity measures**. We compare five different grid sizes: 5.0, 2.0, 1.0 (the original grid size), 0.5, and 0.1 cm by applying different *post-hoc* discretizations of subjects' trajectories—in particular: self-generated patterns **(P)**, letter drawings **(L)**, self-paced random motion **(R1)**, and random motion during pursuit **(R2)**. **(A)** Shows the predictability, **(B)** shows the Lempel Ziv complexity and **(C)** shows the Effective Measure Complexity for all types of subjects' movements and grid sizes.

In order to investigate the impact of the grid size on our results, we performed an additional analysis, where we changed the grid size of our work space *post-hoc*. To compute subjects' motion trajectories for different grid sizes, we discretized the full motion trajectories recorded with a sampling rate of 1 kHz into symbolic sequences with grid sizes 5.0, 2.0, 0.5, and 0.1 cm. The effect of grid size is shown in Figure 7. As expected for smooth trajectories, we found that the finer the grid, the higher the regularity (and predictability) of the different motion types–see Figure 7A. However, if the grid size is chosen extremely coarse (e.g., 5.0 cm), then regularity can increase due to artifacts (e.g., in the case of 5.0 cm there are only two possible transitions from each cell to the next). As the total number of cell transitions increases for smaller cells, the (unnormalized) Lempel-Ziv complexity increases accordingly–see Figure 7B. While the absolute measures of regularity changes across the different grid sizes, importantly, the relative order of regularity between the different motion types remains the same.

The change in Effective Measure Complexity depending on grid size is determined by two factors: one factor is the lowering of the entropy rate for smaller grid sizes due to increased regularity, and the second factor is a faster decay of the conditional entropy for smaller grid size. Depending on the strength of these two factors, Effective Measure Complexity can both increase or decrease. In the case of copying letters, self-paced random movements and random movements in the pursuit game the Effective Measure Complexity increases slightly for smaller grid sizes, mainly due to the effect of the lower entropy rate. In contrast, the Effective Measure Complexity of subjects' self-generated patterns are more sensitive to the change of grid size, and it seems that the structural complexity is highest for a grid size of 2.0 cm –see Figure 7C. Importantly, however, the relative order of the Effective Measure Complexity for the different movement types is robust to changes in grid size.

## Discussion

In our study we designed a motor task to assess complexity and randomness of human hand motion. We analyzed symbol sequences representing subjects' motion trajectories in a discretized workspace. Subjects performed different kinds of movements, including pattern generation, drawing of letters, self-paced random motion, and random movements in the context of a pursuit game. We tested several measures to assess regularity of these movements, including predictability, compressibility, approximate entropy and Lempel-Ziv complexity. We found that all these measures reveal the same order in regularity in these movements, with pattern generation showing the highest degree of regularity, followed by letter drawings, followed by random movements with the lowest degree of regularity. To test whether subjects can adapt their motion randomness, we exposed them to a pursuit game and found that they could increase their randomness in the presence of an on-line Bayesian predictor. However, both subjects' random trajectories before and after training were not fully random compared with synthetic pseudo-random sequences. Finally, we assessed the effective measure complexity of subjects' trajectories as a measure of structural complexity rather than regularity or randomness. We found that self-generated patterns were most complex, followed by letters, followed by random movements that showed the lowest level of complexity.

Previously, Newell et al. (2000) have investigated random motion in single joint finger movements. They instructed subjects to generate random trajectories by varying speed and joint angle of their index finger. The regularity of subjects' movement trajectories was measured by the approximate entropy. The authors found that subjects produced a relatively low level of motion randomness and that subjects were not able to increase the motion randomness significantly through training, even when they provided subjects with additional feedback. In contrast, in our study we found that subjects were able to significantly change the randomness of their movements in the context of a pursuit game compared to self-paced random motion. However, in our study movements were not simple single joint movements, but rather complex multi-joint arm and hand movements required for drawing. Thus, the control process in our experiment has many more degrees of freedom that could be influenced. Similar to Newell et al., we also found that subjects' motion was not fully random and could be easily distinguished from synthetically generated random motion.

In another study, Hornero et al. (2006) asked healthy and schizophrenic subjects to press a space bar on a computer keyboard as irregularly as possible. They assessed the randomness of the resulting time series by approximate entropy, Lempel Ziv complexity and a central tendency measure they specifically developed for this task. The authors found that schizophrenic subjects' time series were characterized by more regularity, that is they were less capable of generating random behavior. Similar to Hornero et al. we found that both approximate entropy and Lempel Ziv complexity provided reliable measures of motion regularity.

While irregularity has been used as a measure of complexity, this is often not the case. In the literature there is a multitude of different complexity measures that can be roughly categorized into three classes (Lloyd, 2001): (i) difficulty of description, (ii) difficulty of creation, and (iii) the degree of organization. The first class typically contains randomness measures such as entropy, Kolmogorov complexity, and Lempel Ziv complexity, with the idea that a symbol sequence that is random is also more difficult to describe. The second class of complexity measures concerns the computational complexity of running an algorithm that can generate a particular sequence and typical measures include time-space computational complexity, logical depth, and others. The third category of complexity aims to quantify the degree of organizational structure and includes effective measure complexity (excess entropy), fractal dimension, hierarchical complexity, and others.

Effective measure complexity is mathematically equivalent to predictive information, that is the mutual information between past and future in a stochastic process (Bialek et al., 2001). It estimates how much information an agent needs to store in its memory so it can predict the future as well as possible after having observed a semi-infinite history (Prokopenko et al., 2009). Intriguingly, predictive information has also previously been suggested as an organizational principle for sensorimotor behavior (Ay et al., 2012; Martius et al., 2013). Rather than maximizing a particular utility function for a particular task, such agents build up efficient representations while exploring their body and environment trying to maximize predictive information.

The estimation of effective measure complexity from finite data is notoriously difficult, because it requires the estimation of higher order block entropies. Estimating entropies of higher order from frequencies in the data introduces a well-known bias that systematically underestimates rare events. There are a number of approaches to correct for this bias (Efron and Stein, 1981; Strong et al., 1998; Antos and Ioannis Kontoyiannis, 2001), but in certain data regimes also “bias-corrected” estimators are likely to be contaminated by bias (Paninski, 2003). Another possibility is to calculate entropy by assuming an underlying Hidden Markov Model (Ekroot and Cover, 1993). However, in our case no such model is available. Another possibility is to estimate entropy from Zipf-ordered frequencies fitted with parametric decay functions (Pöschel et al., 1995). This approach failed in our case, because the parametric families suggested by Pöschel et al. (1995) did not fit the distribution of the motion data very well. In our study we estimated the effective measure complexity based on a decay parameter that we used to interpolate between the empirical conditional entropies and the estimated entropy rates obtained from the normalized Lempel-Ziv complexity. Previous studies showed that the normalized Lempel-Ziv (LZ-76) complexity provides reliable estimates of the entropy rate (Amigó et al., 2004; Speidel et al., 2006). We also found LZ complexity to be robust, when comparing the mean normalized LZ complexity across individuals to the normalized LZ complexity of the concatenated movement sequence of all subjects. The latter symbol sequence had length 100,000 and was comparable to tested sequence lengths in previous studies (Amigó et al., 2004; Speidel et al., 2006).

Based on our estimate of the effective measure complexity we rank-ordered subjects' motion from most complex to least complex from generating patterns, drawing letters, to moving randomly. Drawing letters was characterized both by more randomness and less complexity than subjects' self-generated patterns. One of the reasons for higher irregularity in the letter sequences is certainly that the list of letters subjects were asked to copy was generated randomly from a uniform distribution. Therefore, there was much less repetition than in the case of pattern generation. Moreover, since letters were drawn on top of each other, our recordings include the required connecting movements between different letters. The stereotypy of letters in human perception ignores a lot of the variability and recognition is strongly facilitated by context information and refined feature detection. Both context and perceptual feature spaces are, however, not considered by effective measure complexity, which is a model-free approach to quantify structural complexity.

An important restriction in our analysis is that we only considered spatial patterns by encoding transitions between grid cells independent of the point in time when the transitions occurred. Assessing general spatio-temporal patterns imposes additional challenges: if the temporal resolution is high then changes in the symbol sequence are rare, if the temporal resolution is low then a simple local transition table might not be enough anymore to capture the dynamics, because large spatial jumps can occur. Moreover, the temporal resolution might be different for different subjects, and therefore difficult to assess across subjects. Assessing full spatio-temporal patterns of human movements by symbolic sequences therefore remains an important challenge.

An intriguing question for future research is whether such complexity measures for self-generated motion patterns of different individuals can be associated with personality traits or disease. In particular, it would be interesting to study whether creativity as measured by psychometric approaches can be related to complexity measures of generated motion trajectories. Previous studies have even tried to relate complexity measures of patterns to the degree such patterns are judged to be esthetically pleasing (Ebeling et al., 1998). In the context of motor learning, an interesting question is how structural learning is affected by the structural complexity of different movement types required in different environments (Braun et al., 2009, 2010; Turnham et al., 2012) and how this complexity might affect continuous decision-making processes (Ortega and Braun, 2011; Wolpert and Landy, 2012; Ortega and Braun, 2013). In conclusion, while our study certainly does not provide the final answer to the introductory problem, it suggests that drawing patterns–like for example the Nazca lines in the Peruvian desert or the pictorial engravings in the Voyager Golden Record–might seem not such a bad idea to signal intelligence to an outside observer.

## Materials and methods

### Participants and apparatus

Twenty participants (7 females and 13 males) took part in the study. Participants were assigned into two groups of 10. All participants were naive and gave informed consent before starting the experiment. The study was approved by the ethics committee of the Max Planck Society. We used a virtual reality setup consisting of a Sensable® Phantom® Premium 1.5 High Force manipulandum for tracking participants' hand movements in three dimensions and an NVIS® nVisor ST50 head-mounted display (HMD) for creating stereoscopic 3D virtual reality—see (Genewein and Braun, 2012) for details.

### General experimental procedure

Subjects controlled a cursor (blue, radius 4 mm) representing their hand position in a 3D virtual space. In each trial, their task was to generate a trajectory in the vertical plane in a 10 × 10 cm workspace that was displayed in 3D showing a 10 × 10 grid of unit squares. To initiate the trial they had to move to a start sphere (blue, radius 6 mm) at the center of the square. During the trial, subjects in the first group could see their advancing movement trajectory, as both the current cursor position and all past cursor positions of the trial were displayed. Subjects in the second group (control group) only saw their current cursor position during the trial. This was the only difference between the two groups. Subjects could not move outside the grid as they were constrained to the vertical plane by a spring force (spring constant 8 N/cm) and the boundaries of the grid were delimited by spring-force walls (spring constant 8 N/cm) generated by the manipulandum. Additionally to their three-dimensional hand position, subjects' movements were recorded as transitions in grid space—that is, a state transition was only recorded if the cursor moved to a new grid cell. The trial ended after 200 state transitions. In total, there were five different conditions, each of which consisted of 50 trials. The conditions were organized in two sessions. Session I was a *drawing session* with the three conditions *pattern, letter*, and *random*. Session II was a *pursuit game* with two conditions: *online feedback* and *offline* feedback. The first session was a baseline session in which we could compare randomness and complexity measures of different self-paced movement types, whereas in the second session we could investigate the effect of learning on motion randomness.

#### Drawing session

In the drawing session participants were asked to perform three different types of movement indicated by a written instruction displayed on the screen and condition-specific background colors: *pattern* (gray background), *letter* (black background), and *random* (dark blue background). The first 15 trials were test runs in the order 5 pattern, 5 letter, 5 random. The remaining 150 trials for the three conditions were interleaved randomly. Thus, in total there were 165 trials. For the *pattern* condition, subjects were instructed to “draw something with a repeating pattern.” In each trial, they performed only one such pattern, and they could change the pattern from one trial to the next. The shape of the pattern was not otherwise prescribed, so subjects could pick arbitrary patterns. In the *letter* condition, ten letters were drawn uniformly from the English alphabet and displayed on the screen in a row at beginning of each trial. Subjects were asked to copy them one after another and to write them on top of each other. Subjects were not required to finished all ten letters. In the *random* condition, subjects were asked to “draw trajectories they considered to be random.” A trial ended after 200 cell transitions. Figure 1 shows three examples for the different conditions.

#### Pursuit game

In the pursuit game, an artificial intelligence based on the *Context-Tree Weighting* (CTW) algorithm (Willems, 1995; Volf, 1997) learnt to predict subjects' next move. Context-Trees were learnt on-line across trials through the entire session. Subjects were told that there was an AI predicting their behavior and they “should be as unpredictable as possible.” A test run consisted of the first five trials in the *online feedback* condition, the remaining 100 trials of the two conditions were interleaved randomly. In the *online feedback* condition subjects could see whether their movement position in the last four cells (or respectively the last cell in the control experiment) was predicted by the artificial intelligence or not. If the prediction matched the subject's real move, the cell the subject moved into was marked in red and subjects heard a high pitch beep. If the prediction did not match the subject's real move, the cell the subject moved into was marked blue and no sound was played. Additionally, the fraction of successfully predicted moves in the trial was shown numerically on the screen. A trial ended after 200 cell transitions. In the *offline* feedback condition, subjects did not receive any information about the predictability of their trajectory during the trial, but they could see the fraction of successfully predicted moves at the end of each trial.

### Data analysis

The workspace was divided into 10 × 10 grid cells. Transitions between the grid cells were recorded as symbol sequences *s*_1_*s*_2_*s*_3_…*s*_*n*_, with *s*_*i*_ ∈ {*l, r, u, d*} corresponding to “left,” “right” “up,” and “down.” For each subject, we merged the symbol sequences from all trials of each condition into one big sequence with length *n* = 10,000 (50 trials × 200 transitions per trial).

Additionally, three artificial data sets with the same sequence length (*n* = 10,000) were generated to compare them to the recorded sequences. The first artificial sequence was a repetitive up-down sequence consisting of “*ududududu*…”. The second artificial sequence was a random sequence simulating a particle doing a random walk inside a 10 × 10 grid with uniform transition probabilities. The third artificial sequence was generated by a random process having the same first order frequencies (*p*(*l*), *p*(*r*), *p*(*u*), *p*(*d*)) as subjects' pattern generation sequences. To quantify randomness and complexity of the symbol sequences we used the following measures.

#### Predictability

We quantified the predictability of subjects' motion by counting the number of correctly predicted cell transitions one step into the future given subjects' current position and history. As a predictor we used the *Context-Tree Weighting* (CWT) algorithm (Willems, 1995; Volf, 1997). CWT is an efficient and theoretically well-studied binary sequence prediction algorithm based on online Bayesian model averaging that works well for very general prediction problems (Begleiter et al., 2004; Veness et al., 2011). To apply CWT to ASCII symbol sequences, eight binary sequences are obtained from the ASCII sequence corresponding to the sequences of the first, second, third, etc. up to the eighth bit of each ASCII byte. Therefore, eight context trees for the eight binary sequences are used in parallel. Each tree has a fixed depth *D* that limits the length of the binary patterns the tree can detect. Context trees are binary suffix trees where each pattern (up to length *D*) corresponds to a particular node in the tree. Given a particular node *n*, a prediction tree counts the number of zeros and ones immediately following the pattern (Rissanen, 1983; Ron et al., 1996). At each time point *t*, the probability for the next binary symbol of node *n* in the tree is computed given the full history *S*_1:*t*_ = *S*_1_*S*_2_ ··· *S*_*t*_ of the binary sequence with *a*_*n*_ zeros and *b*_*n*_ ones using the Krichevsky-Trofimov (KT) estimator (Krichevsky and Trofimov, 1981)

(1) $$\begin{array}{l} P_{KT}^{n}(S_{t+1}=0|S_{1:t})=\frac{a_{n}+1/2}{a_{n}+b_{n}+1}\\ P_{KT}^{n}(S_{t+1}=1|S_{1:t})\;=1-P_{KT}^{n}(S_{t+1}=0|S_{1:t}). \end{array}$$

The KT estimator assumes a Bernoulli model, equivalent to observing tosses of heads (zeros) and tails (ones) of a coin with unknown bias and then predicting the most likely next outcome (head or tail corresponding to zero or one). To predict the next symbol of the binary sequence based on this context tree, the weighted probability *P*^ϵ^_*w*_ of the root node ϵ has to be determined, according to the following recursion

(2) $$P_{w}^{n}=\{\begin{array}{ll} P_{KT}^{n}(S_{1:t}) & \text{if }n\text{ is a leaf node}\\ \frac{1}{2}P_{KT}^{n}(S_{1:t})+\frac{1}{2}(P_{w}^{n0}\times P_{w}^{n1}) & \text{otherwise} \end{array}$$

where *P*^*n*0^_*w*_ and *P*^*n*1^_*w*_ represent the weighted probabilities of the left and right child of node *n*. By computing the weighted probability from bottom to top we get the output prediction probability *P*^ϵ^_*w*_ of the next bit in the sequence.

We used the CTW algorithm in two ways. First, we used CTW for on-line prediction in the pursuit game during the experiment. Second, we used CTW as an offline analysis to measure the predictability γ of the symbol sequences obtained from the recorded motion trajectories, where

(3) $$\gamma =n_{m}/N$$

with *n*_*m*_ counting the number of correctly predicted cell transitions and *N* the total length of the sequence.

#### Compressibility

Random sequences are more difficult to compress than regular sequences, because it is difficult to find repeating patterns that could be encoded with short codewords. Therefore, compressibility can also be used to quantify the regularity of sequences and to distinguish between random and non-random sequences. Here we used two compression algorithms for analysis: *Lempel-Ziv* compression and *Context-Tree Weighting* compression.

- *Lempel-Ziv* algorithms compress data by searching for repetitive words, that have appeared before in the sequence. These words are saved in a dictionary such that the sequence can be encoded by the index of the words (Ziv and Lempel, 1978; Welch, 1984). Lempel-Ziv compression is a universal data compression algorithm that does not require prior knowledge of the source statistics and is therefore widely used in practice (Farach and Thorup, 1998).
- *Context-Tree Weighting* cannot only be used for prediction, but also for compression—in fact, it was originally proposed as a lossless compression technique (Willems, 1995). The compressor can be simply obtained by using the CTW predictive distribution as a coding distribution in an arithmetic coding scheme (MacKay, 2003). Computational and storage complexity of this algorithm are linear in the source sequence length.

We define η as a measure of compressibility with

(4) $$\eta =(N-N_{comp})/N,$$

where *N* is the length of the original symbol sequence before compression, and *N*_*comp*_ the length of the compressed sequence. η can also be expressed as a percentage.

#### Approximate entropy

Approximate entropy (ApEn) can quantify the regularity in data without any *a priori* knowledge about the system generating it (Pincus, 1991). It has been used for analyzing regularity in time-series data in neurobiological and other physiological systems (Radhakrishnan and Gangadhar, 1998; Bruhn et al., 2000; Richman and Moorman, 2000; Pincus, 2001; Hornero et al., 2006). ApEn assigns a non-negative value to a time series, with larger values corresponding to more irregularity in the data. It has two user-specified parameters: a run length *m* and a tolerance threshold *r*. For a sequence *s*_1_*s*_2_..*s*_*N*_, ApEn is computed by the following steps (Pincus, 1991; Hornero et al., 2006):

- Form a sequence of vectors *X*_1_, *X*_2_, …, *X*_*N* − *m* + 1_ defined by *X*_*i*_ = [*s*_*i*_, *s*_*i* + 1_, …, *s*_*i* + *m* − 1_].
- Define the distance between *X*_*i*_ and *X*_*j*_ as the maximum absolute difference between their respective scalar components: *d*[*X*_*i*_, *X*_*j*_] = max_*k* = 1:*m*_|*X*_*i*_(*k*) − *X*_*j*_(*k*)| = max_*k* = 1:*m*_ |*s*_*i* + *k* − 1_ − *s*_*j* + *k* − 1_|.
- For each *X*_*i*_ construct a quantity $C_{r}^{m}(i)=\frac{N_{r}^{m}(i)}{N-m+1}$ where *N*^*m*^_*r*_ (*i*) counts the number of *X*_*j*_ such that *d*[*X*_*i*_, *X*_*j*_] < *r*.
- Compute *ϕ*^*m*^_*r*_ by taking the natural logarithm of each *C*^*m*^_*i*_ and averaging it over *i* such that $\phi_{r}^{m}=\frac{1}{N-m+1}\sum_{i\;=\;1}^{N\;-\;m\;+\;1}\ln\;C_{r}^{m}(i)$.
- Repeat the same calculation for *m* + 1 to obtain *ϕ*^*m* + 1^_*r*_.
- Finally ApEn is given by *ApEn*(*m, r*) = *ϕ*^*m*^_*r*_ − *ϕ*^*m* + 1^_*r*_.

Comparisons between time series can only be made given the same values of parameters *r* and *m* (Pincus, 2001). As suggested by previous studies (Pincus, 2001; Hornero et al., 2006), we used the parameter settings *m* ∈ {1, 2} and *r* ∈ {0.1, 0.15, 0.2, 0.25} × σ, where σ is the standard deviation (*SD*) of the symbol sequence in numerical representation. In our case of integer sequences, the estimate of the approximate entropy was not affected by the different parameter settings of *r* ∈ {0.1, 0.15, 0.2, 0.25} × σ.

#### Lempel-Ziv complexity

Lempel-Ziv complexity is a non-parametric entropic measure of regularity of symbol sequences (Doğanaksoy and Göloğlu, 2006). It has been widely applied in neuroscience, for instance, to detect epileptic seizure from EEG data (Radhakrishnan and Gangadhar, 1998; Hu et al., 2006), to analyze neural spike trains (Amigó et al., 2004; Blanc et al., 2008), and to quantify the complexity of states of consciousness (Casali et al., 2013). Roughly, it counts the minimal number of distinct substrings to segment an entire symbol sequence. For instance, the decomposition of the binary sequence *x* = 01001101010111001001 into minimal blocks of the segmentation is 0|1|00|11|0101|0111|0010|01, hence the (LZ-76) complexity of *x* is 8.

#### Effective measure complexity

Effective measure complexity has been proposed as a measure of complexity or structure of a system (Grassberger, 1986; Eriksson and Lindgren, 1987; Lindgren and Nordahl, 1988). It is also referred to as Predictive Information (Prokopenko et al., 2009), Excess Entropy (Crutchfield and Feldman, 2003; Ay et al., 2006), or simply Complexity (Li, 1991). One way to determine effective measure complexity, is to first compute the block-entropy *H*_*L*_ of length-L patterns in the sequence

(5) $$H_{L}=-\sum_{s_{1}\ldots s_{L}}p(s_{1}\ldots s_{L})\log_{k}p(s_{1}\ldots s_{L})$$

where *k* is the size of the alphabet. For example, in a binary sequence *k* = 2 and the block-entropy *H*_*L*_ is measured in bits. In our sequences the alphabet is quaternary consisting of the symbols “*l*,” “*r*,” “*u*,” and “*d*,” and thus *k* = 4. *H*_*L*_ is a non-decreasing function of *L*, which allows defining positive conditional entropies *h*_*L*_ through the difference

(6) $$h_{L}=H_{L+1}-H_{L}$$

sometimes also called *entropy gains* (Crutchfield and Feldman, 2003). The conditional entropy *h*_*L*_ quantifies the average uncertainty about the symbol *s*_*L*+1_ given the previous symbol sequence *s*_1_..*s*_*L*_. The longer the given sequence, the lower the conditional entropy, as adding more prior information can only lead to a better prediction of a symbol, such that *h*_*L*+1_ ≤ *h*_*L*_. The limit *L* → ∞ of the conditional entropy gives the *entropy rate*

(7) $$h=\underset{L\to \infty }{\lim}h_{L},$$

which is also known as per-symbol entropy, the thermodynamic entropy density, Kolmogorov-Sinai entropy (Kolmogorov, 1959), or metric entropy. The entropy rate of a sequence quantifies the average amount of information per symbol *s* and is a lower bound for all conditional entropies such that *h* ≤ *h*_L_ with *L* ∈ ℕ. Therefore, the entropy rate quantifies the amount of irreducible randomness or uncertainty in the system, that is no knowledge of an arbitrarily long sequence preceding a symbol can improve prediction of that symbol beyond this bound.

The decay rate of the conditional entropy with increasing sequence length *L* is an important indicator of complexity. In particular, slowly decaying conditional entropies imply long range order typical for complex systems, because in that case symbols that are far apart in the sequence still share information. These long range correlations allow improving predictability of a symbol when increasing the length of the preceding sequence from *L* to *L* + 1. In contrast, fast decaying conditional entropies are typical for simple systems with no memory. In either case we can think about the conditional entropies as finite approximations to the entropy rate. These finite approximations systematically overestimate the entropy in the system, because part of the entropy vanishes when taking into account longer preceding sequences with larger *L*. Thus, the difference *h*_*L*_ − *h* measures the amount of apparent randomness, that is not really random, but can be explained away by considering correlations over longer distances. Therefore, this local excess entropy *h*_*L*_ − *h* is an indicator of the memory or structure of the source generating the symbol string (Ebeling, 1997; Ebeling et al., 1998).

The total excess entropy is given by the sum over all local excess entropies and corresponds to the *Effective Measure Complexity* (EMC) of the system

(8) $$EMC=\sum_{L\;=\;0}^{\infty }\left(h_{L}-h\right).$$

This quantity converges to a finite value as long as the conditional entropy *h*_*L*_ decays faster than 1/*L*. Simple systems are often characterized by an exponential decay, that is *h*_*L*_ ~ exp(−γ*L*) with some relaxation rate γ > 0. Complex sequences created from music or text have been found to lie between these two extremes and can often be well approximated polynomially (Ebeling and Nicolis, 1991, 1992; Debowski, 2011), such that

(9) $$h_{L}\approx h+\frac{1}{L^{\alpha }},$$

with α ∈ (0;1). The value of α has been found to be usually around 0.5 for text sequences and between 0.5 and 1 for music (Ebeling et al., 1998).

Determining the effective measure complexity from finite data is difficult, especially when estimating the entropies from empirical data frequencies. This requires *L* ≪ log_*k*_ *N*, such that the sequence length *N* is significantly longer than the block lengths *L* to ensure that each symbol combination *s*_1_*s*_2_..*s*_*L*_ occurs sufficiently often. When *L* ≥ log_*k*_ *N*, many strings *s*_1_*s*_2_..*s*_*L*_ will occur only once or not at all, and hence the empirical frequency cannot reflect the underlying distribution anymore. In order to overcome this undersampling problem, we used Equation 9 to approximate the effective measure complexity. Then the problem reduces to estimating the decay parameter α and the entropy rate *h*. The entropy rate can be approximately determined from finite sequences by the normalized Lempel-Ziv complexity, presuming that the source sequence is stationary and ergodic (Lempel and Ziv, 1976; Cover and Thomas, 1991; Badii and Politi, 1999). It provides a straightforward way to estimate the entropy rate for symbolic sequences, requiring no free parameters. Therefore, normalized Lempel-Ziv complexity is a widely used entropy rate estimator in practice (Amigó et al., 2004; Zozor et al., 2005; Amigó and Kennel, 2006). In our study we used the normalization procedure suggested by Badii and Politi in Chapter 8 (Badii and Politi, 1999). Supplementary Figure S1 demonstrates the convergence of the normalized LZ complexity to the true entropy rate (computation details see Ekroot and Cover, 1993) for a random walk in a 10 × 10 grid. The decay parameter α can be fitted when applying Equation 9 to conditional entropies that can still be reliably computed from frequency data, where the condition *L* ≪ log_4_ *N* holds. Finally the estimated Effective Measure Complexity is established by using the approximate conditional entropies for higher order *L* in Equation 8.

### Conflict of interest statement

The authors declare that the research was conducted in the absence of any commercial or financial relationships that could be construed as a potential conflict of interest.
