# Supplementary material for: Assessing randomness and complexity in human motion trajectories through analysis of symbolic sequences
Source: Front Hum Neurosci. 2014 Mar 31;8:168. doi: 10.3389/fnhum.2014.00168 (PMC3978291; doi:10.3389/fnhum.2014.00168)
Supplement: Supplementary file 1 [file DataSheet1.PDF]

Frontiers in Human Neuroscience

Research Article
26 February 2014

1

Supplementary Material: Assessing randomness and complexity in human motion trajectories through analysis of symbolic sequences

Zhen Peng [1,2,3,*], Tim Genewein[1,2,3], Daniel A. Braun[1,2]

[1] *Max Planck Institute for Biological Cybernetics, Tübingen, Germany*
[2] *Max Planck Institute for Intelligent Systems, Tübingen, Germany*
[3] *Graduate Training Centre of Neuroscience, Tübingen, Germany*

Correspondence*:
Zhen Peng
Max Planck Institute for Biological Cybernetics, Spemannstr. 38, 72076 Tübingen, Germany, zhen.peng@tuebingen.mpg.de

1 SUPPLEMENTARY FIGURES

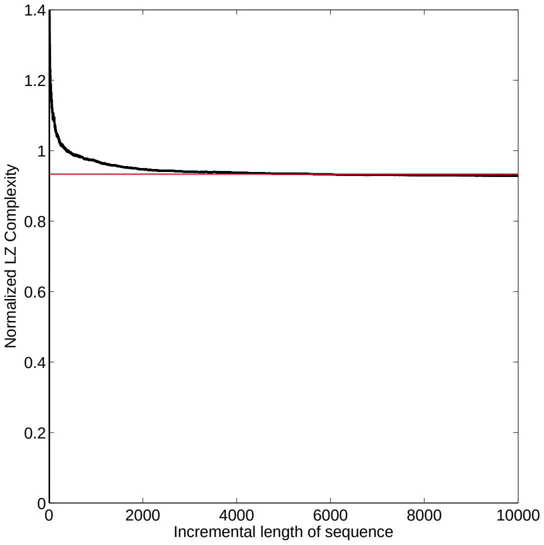

Figure S1. Convergence of normalized Lempel-Ziv complexity towards the entropy rate. The (red) baseline in the figure shows the true entropy rate for a quarternary random process — the process resembles a random-walk inside a 10×10 grid where each grid cell transition is recorded as one of four possible symbols ($s_i \in \{l, r, u, d\}$ corresponding to ‘left’, ‘right’, ‘up’ and ‘down’). The thick (black) line shows the average normalized Lempel-Ziv complexity over 10 random sequences for increasing sequence-length.
